# Short QT Syndrome: A Comprehensive Genetic Interpretation and Clinical Translation of Rare Variants

**DOI:** 10.3390/jcm8071035

**Published:** 2019-07-16

**Authors:** Oscar Campuzano, Anna Fernandez-Falgueras, Ximena Lemus, Georgia Sarquella-Brugada, Sergi Cesar, Monica Coll, Jesus Mates, Elena Arbelo, Paloma Jordà, Alexandra Perez-Serra, Bernat del Olmo, Carles Ferrer-Costa, Anna Iglesias, Victoria Fiol, Marta Puigmulé, Laura Lopez, Ferran Pico, Josep Brugada, Ramon Brugada

**Affiliations:** 1 Cardiovascular Genetics Center, University of Girona-IDIBGI, 17190 Girona, Spain; 2 Centro Investigación Biomédica en Red Enfermedades Cardiovasculares (CIBERCV), 28029 Madrid, Spain; 3 Medical Science Department, School of Medicine, University of Girona, 17071 Girona, Spain; 4 Biochemistry and Molecular Genetics Department, Hospital Clinic, IDIBAPS, 08036 Barcelona, Spain; 5 Arrhythmias Unit, Hospital Sant Joan de Déu, University of Barcelona, 08950 Barcelona, Spain; 6 Arrhythmias Unit, Hospital Clinic, University of Barcelona, 08036 Barcelona, Spain; 7 Cardiology Service, Hospital Josep Trueta, University of Girona, 17007 Girona, Spain

**Keywords:** short QT syndrome, sudden cardiac death, genetics, pathogenicity

## Abstract

Short QT syndrome, one of the most lethal entities associated with sudden cardiac death, is a rare genetic disease characterized by short QT intervals detected by electrocardiogram. Several genetic variants are causally linked to the disease, but there has yet to be a comprehensive analysis of variants among patients with short QT syndrome. To fill this gap, we performed an exhaustive study of variants currently catalogued as deleterious in short QT syndrome according to the American College of Medical Genetics and Genomics and the Association for Molecular Pathology. Analysis of the 32 variants described in the literature determined that only nine (28.12%) have a conclusive pathogenic role. All definitively pathogenic variants are located in *KCNQ1*, *KCNH2*, or *KCNJ2*; three genes encoding potassium channels. Other variants located in genes encoding calcium or sodium channels are associated with electrical alterations concomitant with shortened QT intervals but do not guarantee a diagnosis of short QT syndrome. We recommend caution regarding previously reported variants classified as pathogenic. An exhaustive re-analysis is necessary to clarify the role of each variant before routinely translating genetic findings to the clinical setting.

## 1. Introduction

In 2000, Gussak et al. described short QT syndrome (SQTS) as a new clinical entity. Further, it was described as inherited condition because four individuals from the same family presented with idiopathic and persistent short QT intervals via electrocardiogram (ECG) [1]. In 2011, Gollob et al. proposed a SQTS diagnostic criteria based on four components including ECG, clinical history, family history, and genotype [2]. In 2013, a consensus established that a cutoff value of QTc ≤ 330 ms should be used for the diagnosis [3]. Currently, clinical diagnosis of SQTS is characterized by a shortened QT interval (QTc < 340 ms), tall and peaked T waves, and poor rate adaptation of the QT interval, all without structural heart abnormalities [4]. In addition, SQTS can be also diagnosed with a QTc interval of </= 360 ms with one or more of: (a) a confirmed pathogenic mutation; (b) a family history of SQTS (c) a family history of sudden death at age < 40 years, and (d) survival from a VT/VF episode in the absence of structural heart disease [4].

Individuals with SQTS can exhibit widely varying symptoms, from never experiencing health problems associated with the disease to dizziness, syncope, and even sudden cardiac death (SCD) due to ventricular fibrillation. This malignant arrhythmia can occur any time from early infancy to old age, although most cases occur at a young age. There is also a male predominance to SQTS [5]. The prevalence of SQTS is estimated at 0.02–0.1%; however, this may be underestimated due to no more than 100 cases have been diagnosed worldwide to date [6]. Applying different QTc correction formulae leads to significant reclassification in SQTS probability. The risk of SCD among SQTS patients is high and an implantable cardioverter defibrillator (ICD) may be used to prevent SCD [4]. However, SQTS may be relatively benign if suitable risk assessment and individualized pharmacological measures are applied with early detection [7].

In 2004, Brugada et al. identified the first genetic variant associated with SQTS, located in the *KCNH2* gene [8]. Additional potentially pathogenic variants have been reported in five genes (*CACNA2D1, KCNH2, KCNJ2, KCNQ1*, and *SLC4A3*); all exhibit autosomal dominant inheritance. Comprehensive genetic analysis is used to identify the cause of SQTS in nearly 20% of diagnosed cases. However, this percentage may be misleading due to the low number of reported families. This lack of families also impedes a robust genotype–phenotype correlation assessment for risk stratification [4]. Genetic variants have been reported in three other genes: *CACNA1C, CACNB2b*, and *SCN5A*, although individuals with these variants show a characteristic phenotype of Brugada syndrome (BrS) with concomitant shortened QT intervals, but without a conclusive clinical diagnosis of SQTS. Despite controversial data, current guidelines recommend genetic analysis of five genes: *KCNH2, KCNQ1, KCNJ2, CACNA1C*, and *CACNB2b*) in the diagnosis of SQTS [4]. Family assessment is mandatory when a patient is diagnosed via genetic testing, allowing for the early identification of relatives at risk [9].

In 2015, the American College of Medical Genetics and Genomics and the Association for Molecular Pathology (ACMG/AMP) published recommendations for the interpretation of rare variants, hoping to clarify their pathogenic role [10]. Each entry describes evidence for both pathogenicity and benignity to enable the classification of variants as pathogenic, likely pathogenic, variants of uncertain significance, likely benign, and benign. The Human Genome Mutation Database (HGMD) classifies a few variants as disease-causing mutations and potentially damaging associated with SQTS, although these variants have not been comprehensively examined following ACMG/AMP recommendations. However, variants’ pathogenicity classifications are based on limited evidence. Therefore, we performed a comprehensive analysis of all published data to clarify the roles of rare variants currently reported as potentially deleterious in SQTS and to establish the genes associated with conclusive clinical diagnosis of SQTS.

## 2. Experimental Section

Data concerning “Short QT syndrome and/or Short QT interval” was collected from: HGMD (http://www.hgmd.org), ClinVar (https://www.ncbi.nlm.nih.gov/clinvar/intro/), the National Center for Biotechnology Information SNP database (http://www.ncbi.nlm.nih.gov/SNP), Index Copernicus (http://en.indexcopernicus.com), Google Scholar (https://scholar.google.es), Springer Link (https://link.springer.com), Science Direct (https://www.sciencedirect.com), Excerpta Medica Database (https://www.elsevier.com/solutions/embase-biomedical-research), and the IEEE Xplore Digital Library (http://ieeexplore.ieee.org/Xplore/home.jsp).

Genetic variants identified in articles were contrasted with variant data from HapMap (http://hapmap.ncbi.nlm.nih.gov), 1000 Genomes Project (http://www.1000genomes.org), Exome Variant Server –EVS- (http://evs.gs.washington.edu/EVS), Genome Aggregation Database -gnomAD- (http://gnomad.broadinstitute.org/), and Exome Aggregation Consortium -ExAC- (http://exac.broadinstitute.org), including recently added data concerning copy number variations (CNV). Concerning global frequencies in population databases, it is important to remark that ethnic differences should be taking into account in a final classification. In addition, we consulted data concerning amino acid structure, including Grantham score and MutPred (http://mutpred.mutdb.org). An exhaustive in silico prediction of pathogenicity of genetic variations also was assessed using Combined Annotation Dependent Depletion -CADD- (http://cadd.gs.washington.edu), Genomic Evolutionary Rate Profiling -GERP- (http://mendel.stanford.edu/SidowLab/downloads/gerp), Mendelian Clinically Applicable Pathogenicity Score -M-CAP- (http://bejerano.stanford.edu/mcap), Fathmm-MKL (http://fathmm.biocompute.org.uk/fathmmMKL.htm), MetaSVM and MetaLR (which represents two ensemble methods that combine 10 predictor scores: SIFT, PolyPhen-2 HDIV, PolyPhen-2 HVAR, GERP, MutationTaster, Mutation Assessor, FATHMM, LRT, SiPhy, and PhyloP), Mutation Taster (http://www.mutationtaster.org), Mutation Assessor (http://mutationassessor.org), Phylogenetic Analysis -PhyloP- (http://compgen.cshl.edu/phast/background.php), Protein Variation Effect Analyzer -PROVEAN- (http://provean.jcvi.org/index.php), and Polymorphism Phenotyping v2 -PolyPhen2, PPH2- (http://genetics.bwh.harvard.edu/pph2).

Variants were classified according to ACMG/AMP standards and guidelines for the interpretation of sequence variants (ACMG classification) [10]. Hence, ACMG/AMP recommendations describe several items of pathogenicity (PVS: Evidence of Pathogenicity Very Strong, PS: Evidence of Pathogenicity Strong, PM: Evidence of Pathogenicity Moderate, and PP: Evidence of Pathogenicity Supporting), and benignity (BA: Evidence of benign impact Stand-alone, BS: Evidence of benign impact Strong, and BP: Evidence of benign impact Supporting), enabling and a final score and consequent classification of variants in P: Pathogenic, LP: Likely Pathogenic, VUS: Variant of Uncertain Significance, LB: Likely Benign and B: Benign. The PM2 item in the ACMG classification was considered fulfilled if minor allele frequency (MAF) in relevant population databases was ≤0.1% [11]. Concerning frequency of disease-causing variants, vast majority of pathogenic variants are extremely rare (<0.01%) [12]. Concerning PVS1, it should only be used for variants in genes where loss of function is a previously established disease mechanism (https://www.ncbi.nlm.nih.gov/projects/dbvar/clingen/) [13]. In addition, some items of ACMG/AMP may underlie a lack of specificity or ambiguous or contradictory interpretations, so we check the parameters using Sherloc (semiquantitative, hierarchical evidence-based rules for locus interpretation) [14]. Three authors independently conducted a comprehensive investigation of published data concerning each variant analyzed in our study. All investigators discussed data included in each item of the ACMG, and consensus a final classification of all variants in order to avoid any bias.

## 3. Results

Published data concerning SQTS show 15 variants reported as disease mutations (DM): two in *KCNQ1*, eight in *KCNH2*, three in *KCNJ2*, and one variant in *SLC4A3*. In addition, 17 variants have been classified as having ambiguous significance due to a lack of conclusive data and conflicting clinical phenotypes. These rare variants have been identified in five genes: *KCNQ1, KCNJ2, CACNA1C, CACNB2*, and *SCN5A*. Therefore, rare variants potentially associated with SQTS or similar phenotypes are reported in eight genes: *CACNA1C, CACNA2D1, CACNB2, KCNH2, KCNJ2, KCNQ1, SCN5A*, and *SLC4A3*.

### 3.1. The CACNA2D1 Gene

**Figure 1:**
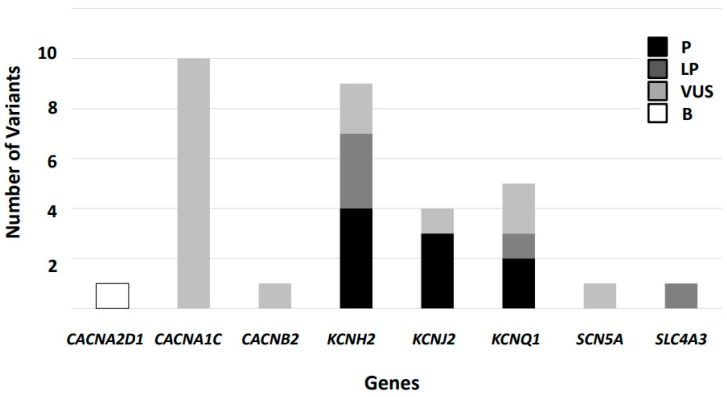
Classification of variants reported in Short QT Syndrome following American College of Medical Genetics and Genomics/Association for Molecular Pathology guidelines. Thirty-two variants were identified but only nine remains as definitively deleterious. P: Pathogenic, LP: Likely Pathogenic, VUS: Variant of Uncertain of Significance, B: Benign.

Only one variant classified as pathogenic for SQTS -p.(Ser755Thr), rs151327713, CM111612- has been reported [15]. Other reports identified the same rare variant in a patient diagnosed with BrS, and functional in vitro studies showed no significant ion current alterations in mutant cells in comparison to controls. The variant is now identified in low frequencies (Table 1). ClinVar classifies the variant as having ambiguous significance. In addition, in silico databases show a contradictory role for this variant (Table 2). Considering all data, p.S755T should be classified as Benign for SQTS following ACMG/AMP recommendations (Table 1 and Table 3, Figure 1).

### 3.2. Other Variants Potentially Associated with Short QT Syndrome in Calcium Channels

In 2007, three probands with a BrS phenotype and short QT intervals were published. Genetic analysis identified *missense* variants in *CACNA1C*: p.(Ala39Val) -CM070048-, p.(Gly490Arg) and *CACNB2*: p.(Ser481Leu). In vitro studies confirmed loss-of-function in calcium channel activity in p.(Ala39Val) [16] but Simms and Zamponi did not identify functional effects [17], although this last manuscript used the neuronal Cav1.2, not the cardiac isoform. Considering all data, including contradictory in silico predictions (Table 2), p.(Ala39Val) should be classified as VUS for SQTS following ACMG/AMP recommendations (Table 1 and Table 3, Figure 1). The p.(Gly490Arg) variant (rs121912775, CM070047) in *CACNA1C* is identified in low frequencies. The variant is associated with LQTS and BrS, playing an ambiguous role. This contradictory effect is supported by differing in silico predictions (Table 2). The p.(Gly490Arg) variant should be classified as VUS for SQTS following ACMG/AMP recommendations (Table 1 and Table 3, Figure 1).

Five rare variants in patients showing BrS and shorter than normal QT intervals were reported in the *CACNA1C* gene [18]. The first variant p.(Glu1115Lys) -rs199473391, CM109282- has not been identified in global databases so far. However, the same rare variant was recently identified in a patient showing an enlarged QT interval [19]. Considering all data, including contradictory in silico predictions (Table 2), p.(Glu1115Lys) should be classified as VUS for SQTS following ACMG/AMP recommendations (Table 1 and Table 3, Figure 1). The variant p.(Glu1829_Gln1833dup) -CI109266- was identified in a phenotype of BrS with a shorter than normal QT interval. It has not been identified in global databases and in silico databases predict a deleterious effect (Table 2). Considering all data, p.(Glu1829_Gln1833dup) should be classified as VUS for SQTS following ACMG/AMP recommendations (Table 1 and Table 3, Figure 1). The third variant was p.(Arg1880Gln) -rs182208896, CM109283-. It has not been identified in global databases but was identified in other reports highlighting the need for a cautious clinical interpretation. Due to conflicting information, including contradictory in silico prediction (Table 2), p.(Arg1880Gln) should be classified as VUS for SQTS following ACMG/AMP recommendations (Table 1 and Table 3, Figure 1). The fourth variant was p.(Val2014Ile) (rs199473660, CM109284). In global databases has not been identified but it was identified in other reports highlighting a cautious interpretation, and in silico databases show a contradictory role (Table 2). Considering all divergent data, p.(Val2014Ile) should be classified as VUS for SQTS following ACMG/AMP recommendations (Table 1 and Table 3, Figure 1). Finally, the fifth variant was p.(Asp2130Asn) (rs199473392, CM109285). Due to conflicting information, including contradictory in silico prediction (Table 2), p.(Asp2130Asn) should be classified as VUS for SQTS following ACMG/AMP recommendations (Table 1 and Table 3, Figure 1).

In 2013, three more variants were identified in *CACNA1C* in patients showing BrS and shorter than normal QT intervals [20]. The first variant was p.(Asn547Ser) -rs768614762, CM133491-. In global populations, it has been identified at low frequency and in silico databases show a conflicting role (Table 2). Considering all incongruous data, p.(Asn547Ser) should be classified as VUS for SQTS following ACMG/AMP recommendations (Table 1 and Table 3, Figure 1). The second variant was p.(Arg632Arg) -CS133492-. It produces no amino acid change, but the codon 1896 is the first nucleotide of exon 14 and causes a splicing error. Such a splicing error results in an exon skipping and a frame-shift, thereby a premature termination codon, which in turn causes the nonsense mutation-mediated decay of mRNA (NMD). However, the corrected QT interval was within the normal range (383 ms). In 2014, the same group reported that mutant mRNA with a c.(1896G > A) substitution may be diminished by nonsense-mediated mRNA decay [21]. Due to conflicting information, including in silico prediction (Table 2), p.(Arg632Arg) should be classified as VUS for SQTS (Table 1 and Table 3, Figure 1). The third variant was p.(Arg1780His) (rs756829999, CM133493). Due to conflicting information reported so far, p.(Arg1780His) should be classified as VUS for SQTS following ACMG/AMP recommendations (Table 1 and Table 3, Figure 1).

Finally, the *CACNB2_*p.(Ser481Leu) variant (CM070049) was reported in a family showing BrS phenotype and short QT interval [16]. In vitro studies confirmed loss-of-function in calcium channel activity but in silico databases show a contradictory role (Table 2). Considering all divergence data, p.(Ser481Leu) should be classified as VUS for SQTS following current ACMG/AMP recommendations (Table 1 and Table 3, Figure 1).

### 3.3. The KCNH2 Gene

In 2003, three families with several members diagnosed with SQTS were reported [22]. The p.(Asn588Lys) variant (rs104894021, CM040082/CM040083) was identified in *KCNH2*. Conclusive data concerning pathogenicity was recently reported [23], even using human-induced pluripotent stem cell–derived cardiomyocytes (hiPSC-CMs) [24,25] supporting a deleterious role previously proposed in 2003. Considering all data, including in silico analysis (Table 2), p.(Asn588Lys) should be classified as Pathogenic for SQTS following ACMG/AMP recommendations (Table 1 and Table 3, Figure 1).

In 2008, a novel variant in *KCNH2*, p.(Arg1135His) -rs199473547, CM086664- was reported [26]. Functional studies showed a potential repolarization effect but no additional studies have been reported. Considering all data, including contradictory in silico prediction (Table 2), p.(Arg1135His) should be classified as VUS for SQTS following ACMG/AMP recommendations (Table 1 and Table 3, Figure 1).

One additional rare variant, p.(Glu50Asp) -rs199472841, CM094307-, was reported [27]. It was previously classified as pathogenic despite no familial segregation, based on in silico predictions (Table 2). Considering all data, p.(Glu50Asp) should be classified as Likely Pathogenic for SQTS following ACMG/AMP recommendations (Table 1 and Table 3, Figure 1) due to lack of conclusive studies concerning pathogenicity in SQTS. In 2011, several relatives showing SQTS were reported [28]. Genetic analysis identified the p.(Thr618Ile) variant (rs199472947, CM111008) in *KCNH2*. Functional analysis identified a mechanism for its potential pathogenic role [29]. Recently, Guo et al. published a study using human-induced pluripotent stem cell–derived cardiomyocytes (hiPSC-CMs), facilitating our understanding of the mechanism of the disease and confirming its deleterious role in SQTS [30].Considering all published data, including in silico prediction (Table 2), p.(Thr618Ile) should be classified as Pathogenic for SQTS following ACMG/AMP recommendations (Table 1 and Table 3, Figure 1).

In 2014, an additional rare variant -p.(Arg164Cys), CM141125- was reported in a cohort of BrS patients [31]. No additional studies have been published to date. Considering all data, including incongruous in silico prediction (Table 2), p.(Arg164Cys) should be classified as VUS for SQTS following ACMG/AMP recommendations (Table 1 and Table 3, Figure 1). The same report of Wang et al. also identified the p.(Trp927Gly) variant (CM141126) in *KCNH2* as associated with SQTS Despite one publication reporting its pathogenicity, no additional studies support this role. Considering all published data, including in silico prediction (Table 2), p.(Trp927Gly) should be classified as Likely Pathogenic for SQTS (Table 1 and Table 3, Figure 1).

In 2015, another variant in *KCNH2* p.(Ile560Thr) -rs372725107, CM156034- was identified in a SQTS patients as pathogenic. This role is supported by a recent publication [32]. Considering all data, p.(Ile560Thr) should be classified as Pathogenic for SQTS following ACMG/AMP recommendations, despite no familial phenotype–genotype segregation (Table 1 and Table 3, Figure 1).

Recently, a novel variant in *KCNH2*, p.(Ser631Ala), was identified in a family with several relatives who died suddenly at young ages [33]. None of those who died were diagnosed with SQTS but three living relatives were diagnosed and carried the same novel genetic variant -*KCNH2*_p.(Ser631Ala)-. In 2018, a functional study was published concerning this rare variant showed deleterious in vitro role [34]. Considering all data, including in silico prediction (Table 2), p.(Ser631Ala) should be classified as Likely Pathogenic for SQTS (Table 1 and Table 3, Figure 1).

### 3.4. The KCNJ2 Gene

The first reported variant associated with SQTS in *KCNJ2* was p.(Asp172Asn) -rs104894584, CM051549-. Familial genetic analysis identified the variant in two clinically affected relatives [35]. Functional analysis provided evidence for pro-arrhythmic effects in perpetuating and facilitating re-entrant excitation waves. Considering all published data, including in silico predictions (Table 2), p.(Asp172Asn) should be classified as Pathogenic (Table 1 and Table 3, Figure 1).

In 2012, the p.(Met301Lys) variant -CM1110289- was reported [36]. A second publication identified p.(Met301Lys) in a young patient with juvenile-onset atrial fibrillation who also carried the rare genetic variant p.(Gly299Asp) in *KCNQ1*, which was considered the cause of disease [37]. Considering all data, including in silico predictions (Table 2), p.(Met301Lys) should be classified as Pathogenic for SQTS following ACMG/AMP recommendations, despite no familial segregation (Table 1 and Table 3, Figure 1).

The third variant, p.(Glu299Val) -rs786205817, CM131839-, was reported in 2013 [38]. Considering all data, including in silico predictions (Table 2), p.(Glu299Val) should be classified as Pathogenic for SQTS (Table 1 and Table 3, Figure 1).

### 3.5. Other Variants in KCNJ2 Potentially Associated with Short QT Syndrome

In 2014, monozygotic twins displaying short QT intervals and autism-epilepsy syndrome were reported [39]. Genetic analysis identified a novel variant in *KCNJ2*, p.(Lys346Thr) -CM148325-. Considering all data, p.(Lys346Thr) should be classified as VUS for SQTS following ACMG/AMP recommendations (Table 1 and Table 3, Figure 1).

### 3.6. The KCNQ1 Gene

Two variants associated with SQTS and classified as DM were identified in *KCNQ1*: p.(Val307Leu) -rs120074195, CM041383- [40], and p.(Phe279Ile) -CM157723- [41]. Functional and in silico simulation of p.(Val307Leu) supported a pathogenic role (Table 2). Considering all data p.(Val307Leu) should be classified as Pathogenic for SQTS following ACMG/AMP recommendations, despite lack of familial segregation (Table 1 and Table 3, Figure 1). For p.(Phe279Ile), all data support a Likely Pathogenic role for SQTS, despite the lack of familial segregation (Table 1 and Table 3, Figure 1).

### 3.7. Other Variants in KCNQ1 Potentially Associated with Short QT Syndrome

In 2005, a novel in utero diagnosis characterized by atrial fibrillation and SQTS was reported. Genetic analysis identified a de novo variant in *KCNQ1*, p.(Val141Met) -CM056972- [42]. Functional studies showed an alteration in function [43]. Posterior studies confirmed the curious phenotype in patients carrying the same variant [44,45]. Considering all data, including in silico predictions (Table 2), p.(Val141Met) should be classified as Pathogenic for SQTS following ACMG/AMP recommendations (Table 1 and Table 3, Figure 1).

Another variant in *KCNQ1*, p.(Ile274Val) -rs199472728, CM070181-, was identified in a sudden infant death syndrome (SIDS) case [46]. This variant was identified with a frequency >0.3%, and in silico databases show a contradictory role (Table 2). Considering all data, p.(Ile274Val) should be classified as VUS for SQTS (Table 1 and Table 3, Figure 1).

In 2006, the *KCNQ1_*p.(Arg259His) variant (rs199472720, CM064074) was reported in a LQTS patient [47]. An additional report confirmed its deleterious effect in vitro [48], and in silico databases show a contradictory role (Table 2). Considering all data, p.(Arg259His) should be classified as VUS for SQTS (Table 1 and Table 3, Figure 1).

### 3.8. The SCN5A Gene

In 2004, Ackerman et al. analyzed apparently healthy individuals and identified the variant p.(Arg689His) -rs199473145, CM057209- [49]. One year later, the same variant was identified in a patient diagnosed with LQTS [50]. Further, Nakajima et al. identified the variant in a cohort of patients diagnosed with BrS [51]. In 2012, a publication identified the same rare variant in an asymptomatic 40-year-old male with family history of SD who had a Brugada-like ECG with short QT intervals [52]. Other reports have identified the same rare variant in global populations. Due to conflicting information, including frequencies and in silico databases (Table 2), p.(Arg689His) should be classified as VUS for SQTS following ACMG/AMP recommendations (Table 1 and Table 3, Figure 1).

### 3.9. The SLC4A3 Gene

In 2017, a novel variant p.(Arg370His) in *SLC4A3* was reported as associated with SQTS [53]. This variant (CM1717443) was identified in several clinically affected relatives of two families. Considering all published data (Table 2), p.(Arg370His) should be classified as Likely Pathogenic for SQTS (Table 1 and Table 3, Figure 1).

## 4. Discussion

Pathogenic role of rare variants has been debated in almost all diseases areas but especially controversial in SQTS given the immediate impact of the lethality and reduced penetrance of many variants even in well established genes. Nowadays, 32 rare variants have been reported with a potential causative role in SQTS. These variants are reported in HGMD, ClinVar and PubMed. First two databases only archives submitted information available about a variant or condition from other public resources. In addition, both HGMD and ClinVar databases neither curates content and modifies interpretations independent of an explicit submission. Therefore, some of variants currently interpreted as DM may lead to confusion in clinical translation. One of parameters used in the published reports is in silico prediction; it is important to note that these algorithms are only mathematical approaches and are recognized to be poor predictors of diseases causation. An in silico prediction cannot faithfully reflect a human mechanistic pathway. Therefore, can be a marked divergence between in silico predictions and functional data, as we previously reported, and the use of bioinformatic tools can produce erroneous conclusions regarding pathogenicity [54]. Hence, despite included in ACMG/AMP recommendations, they represent the lowest level of evidence for pathogenicity classification. In consequence, a comprehensive analysis of all data published may help to clarify the real role of rare variants currently reported as potentially deleterious in SQTS, and, in addition, establish the main genes associated with conclusive clinical diagnosis of SQTS. In recent years, use of hiPSC-CMs help to solve this ambiguous pathogenic role, at least in large part of analyzed rare variants [24]. Therefore, hiPSC-CMs are able to recapitulate the single-cell phenotypic features of SQTS and provide novel opportunities to further elucidate the disease mechanism and test drug effects [25].

Current guidelines published in 2015 recommend analysis of five genes (*KCNH2, KCNQ1, KCNJ2, CACNA1C*, and *CACNB2b*) in suspected SQTS cases [4]. Concerning calcium genes, all variants reported so far are associated with patients showing BrS and shorter QT intervals, but not a conclusive clinical diagnosis of SQTS. This is an important point in the final clinical classification because of a variant could be pathogenic for a phenotype showing a reduction of the QT interval but not SQTS as entity. The *CACNA2D1* gene was also reported associated with concomitant alterations in suspected SQTS cases but comprehensive analyses conclude a benign role of the reported variant; therefore, it not should be included in the recommended analysis. Similar situation occurs with rare variant in the *SCN5A* gene; not conclusive diagnosis is established so no definite association gene-disease is currently reported. The same situation occurs with *SLC4A3* and its association with SQTS; no conclusive gene-disease relation has been reported so far. Hence, this new candidate gene should not be included in a comprehensive genetic analysis of patients with a conclusive diagnosis of SQTS. Therefore, considering all data reported so far, we believe that analysis of three genes (*KCNH2, KCNQ1*, and *KCNJ2*) should be performed in all suspected cases of SQTS, followed by a comprehensive genetic interpretation of each rare variant identified before translation into clinical practice. Gene-disease association is an important point to consider before clinical translation and, in concordance to current available data concerning other genes (*CACNA1C, CACNA2D1, CACNB2b, SCN5A*, and *SLC4A3*), they should not be analyzed in SQTS cases. Misinterpretation of the diagnosis is a current problem on SQTS. Therefore, several rare variants have been reported in families showing border QTc or Brugada-like ECG with short QT intervals, but not a certain SQTS. Performing an accurate clinical assessment in order to acquire (or not) a conclusive SQTS diagnosis of patients is crucial before genetic analysis but also in adoption of personalized suitable measures for prevention of SCD.

### 4.1. Variants Reported as Associated with Short QT Syndrome

Our comprehensive analysis of the only known *CACNA2D1* variant, p.(Ser755Thr), revealed a benign role. This classification is due to conflicting data concerning phenotypes because it is associated with a BrS-like phenotype with concomitant shorter QT intervals. In *KCNH2*, nine variants are currently reported as DM, but data concerning global frequency along with in silico and functional analyses confirm a definitively pathogenic role of only four variants: p.(Asn588Lys) c.(1764C>A), p.(Asn588Lys) c.(1764C>G), p.(Thr618Ile), and p.(Ile560Thr). Three variants, p.(Glu50Asp), p.(Ser631Ala) and p.(Trp927Gly), were classified as Likely Pathogenic due to a lack of functional data as well as family segregation. Another two variants in *KCNH2*, p.(Arg1135His) and p.(Arg164Cys), remain of ambiguous significance because of both were identified in patients showing BrS and shortened QT intervals, but did not result in a definite clinical diagnosis of SQTS. Of the three variants in *KCNJ2* associated with SQTS so far, all three variants -p.(Met301Lys), p.(Glu299Val) and p.(Asp172Asn)- are classified definitively as Pathogenic, and all current published data support a deleterious role in SQTS. In *KCNQ1*, one variant, p.(Val307Leu), remains classified as Pathogenic and the other variant, p.(Phe279Ile), is currently classified as Likely Pathogenic. Finally, the potential pathogenic variant p.(Arg370His) reported in the *SLC4A3* gene supposes the potential association of a new gene with SQTS but also represents a previously unappreciated mechanism for the development of the malignant arrhythmia. Notably, all carriers had a QTc ≤ 370 ms (not 340 ms, accordingly to current clinical recommendations) supporting the no conclusive gene-disease association. In concordance to this no-conclusive association, functional studies were performed in zebrafish model; therefore, further studies should be performed in order to conclude a definite association.

It is important to remark that despite pathogenic role reported for some rare variants, clinical translation should be done with caution and personalized genetic interpretation is necessary; this fact is especially essential for rare variants without conclusive familial segregation.

### 4.2. Variants Reported as Potentially Associated with Short QT Syndrome

In all consulted databases, sixteen variants in five genes were potentially associated with SQTS but had inconclusive data to make a final determination. Except for one, *KCNQ1_*p.(Val141Met), we recommend that these variants remain designated as having no conclusive relation to SQTS, though they may be implicated in phenotype modulation.

All eleven variants in *CACNA1C* and *CACNB2* have been associated with BrS and shorter QT intervals, but their roles in SQTS remain ambiguous (classified as VUS) due to lack of conclusive clinical data. No patient carrying a variant in either calcium gene has been associated with a conclusive diagnose of SQTS. In addition, the variant *CACNA1C*_p.(Gly490Arg) has been identified in both LQTS and BrS cohorts. To our knowledge, nowadays there are no reports showing that a sole pathogenic variant cause two different pathologies in any human disease.

Concerning potassium genes, only one variant in *KCNJ2*_p.(LysK346Thr) had been reported as potentially causal in SQTS but published data reveal an ambiguous role. This is mainly due to a characteristic phenotype of autism and epilepsy with a concomitant short QT interval [39]. Although there is no conclusive association with SQTS, the authors of that previous study suggest a neuropsychiatric evaluation in patients with short QT intervals who carry variants in this potassium gene. The p.(Val141Met) variant in *KCNQ1* was associated with a novel entity characterized by SQTS but concomitant with other clinical alterations. Curiously, HGMD classifies the variant as DM associated with atrial fibrillation, but no known current studies support this association. Due to a large number of reported cases associating this variant with SQTS, it is classified as definitively Pathogenic in SQTS, playing a deleterious role in clinical practice. The p.(Ile274Val) variant in *KCNQ1* was identified in a SIDS cohort. Due to difficult characterization of death in SIDS, and a lack of functional evidence as well as family segregation, prudence should be taking into consideration before clinical translation [55]. Therefore, it remains classified as being of uncertain significance in SQTS. Finally, the p.(Arg259His) variant in *KCNQ1* remains deleterious in LQTS due to identification in cohorts experiencing this arrhythmogenic disease. However, a solitary report identified a SQTS patient carrying this same variant [56]. Considering the reported contradictory data concerning clinical phenotype, this variant should be classified as VUS in both arrhythmogenic entities.

Finally, one variant of ambiguous significance in SQTS is reported in *SCN5A*, a gene associated with BrS but also responsible for nearly 10% of LQTS cases [57]. The p.(Arg689His) variant has been identified in reports analyzing both LQTS and BrS patients. The same variant was identified in a case with Brugada-like ECG and short QT intervals. To our knowledge, there are no existing descriptions of a sole variant causing two different pathologies, as already mentioned. Considering all published data, this rare variant is classified as VUS for SQTS phenotypes.

A limitation of this study is the lack of reported information for some analyzed variants concerning functional studies as well as familial segregation, which are crucial pieces in the final translation into the clinical setting. It is important to remark that all predictions were performed according to current published data, but additional data may be available in the future. ACMG/AMP classification incorporates a vast number of elements to establish pathogenicity of the variant, despite the lack of validation in large study cohorts. Our classification was designed to have general applicability but also requires further functional studies for gene variant screenings to be correctly applied.

## 5. Conclusions

In conclusion, a limited number of SQTS variants have been classified as potentially pathogenic despite a lack of comprehensive classification following recent ACMG/AMP recommendations. Our comprehensive analysis of all published data for these variants revealed that only 28.12% of reported variants have a conclusive lethal role in SQTS. Importantly, variants with a definite deleterious role are located only in genes encoding potassium channels (*KCNQ1, KCNH2*, and *KCNJ2*). Rare variants located in genes encoding calcium and sodium channels are associated with the concomitant phenotype of BrS and slightly reduced QT interval but not an explicit diagnosis of SQTS. We recommend that care be taken with all previously reported variants classified as pathogenic and that the potassium channel variants be the only genetic clues used to diagnose SQTS. An exhaustive re-analysis is necessary to clarify the actual role of each variant related to SQTS before clinical translation is possible.

## Figures and Tables

**Table 1:** Pathogenic and additional variants associated with Short QT Syndrome.

Gene	Protein	dbSNP	EVS MAF (%) EA/AA/All	ExAC Alleles (%)	gnomAD Alleles (%)	HGMD (DM)	ClinVar	ACMG/AMP Classification
*CACNA1C*	p.(Ala39Val)	rs121912776	-	-	-	BrS + stnQT	P	VUS
	p.(Gly490Arg)	rs121912775	-	52/64,254 (0.08)	154/259,550 (0.059)	VUS (BrS + stnQT)	P	VUS
	p.(Asn547Ser)	rs768614762	-	1/53,304 (0.001)	-	BrS + stnQT	VUS	VUS
	p.(Arg632Arg)	-	-	-	-	BrS + stnQT	-	VUS
	p.(Glu1115Lys)	rs199473391	-	-	-	BrS + stnQT	VUS	VUS
	p.(Arg1780His)	rs756829999	-	-	3/239226 (0.0012)	BrS + stnQT	VUS	VUS
	p.(Glu1829_Gln1833dup)	-	-	-	-	BrS + stnQT	-	VUS
	p.(Arg1880Gln)	rs182208896	-	59/120,512 (0.048)	166/276,890 (0.059)	VUS (BrS + stnQT)	VUS	VUS
	p.(Val2014Ile)	rs199473660	-	-	91/268,760 (0.033)	VUS (BrS + stnQT)	VUS	VUS
	p.(Asp2130Asn)	rs199473392	-	-	29/237,712 (0.012)	BrS + stnQT	VUS	VUS
*CACNA2D1*	p.(Ser755Thr)	rs151327713	0.1047/0.0/0.0692	93/120,134 (0.07)	233/275,628 (0.084)	CM111612	VUS	B
*CACNB2*	p.(Ser481Leu)	-	-	-	-	BrS + stnQT	-	VUS
*KCNH2*	p.(Glu50Asp)	rs199472841	-	-	-	CM094307	VUS	LP
	p.(Arg164Cys)	-	-	-	1/30828 (0.0032)	CM141125	-	VUS
	p.(Ile560Thr)	-	-	-	-	CM156034	-	P
	p.(Asn588Lys) c.(1764C>A)	rs104894021	-	-	-	CM040083	P	P
	p.(Asn588Lys) c.(1764C>G)	rs104894021	-	-	-	CM040082	P	P
	p.(Thr618Ile)	rs199472947	-	-	-	CM111008	VUS	P
	p.(Ser631Ala)	-	-	-	-	-	-	LP
	p.(Trp927Gly)	-	-	-	-	CM141126	-	LP
	p.(Arg1135His)	rs199473547	-	-	2/30,890 (0.0064)	CM086664	VUS	VUS
*KCNJ2*	p.(Asp172Asn)	rs104894584	-	-	-	CM051549	P	P
	p.(Glu299Val)	rs786205817	-	-	-	CM131839	LP	P
	p.(Met301Lys)	-	-	-	-	CM1110289	-	P
	p.(Lys346Thr)	-	-	-	-	Epilepsy + stnQT	-	VUS
*KCNQ1*	p.(Phe279Ile)	-	-	-	-	CM157723	-	LP
	p.(Val307Leu)	rs120074195	-	-	-	CM41383	P	P
	p.(Val141Met)	-	-	-	-	SQTS + AF	-	P
	p.(Ile274Val)	rs199472728	0.0116/0.0/0.0077	39/119,484 (0.03)	49/276,714 (0.0177)	SIDS	LP (LQTS, SIDS)	VUS
	p.(Arg259His)	rs199472720	-	1/104,552 (0.0009)	5/271,834 (0.0018)	LQTS	LP/P (LQTS)	VUS
*SCN5A*	p.(Arg689His)	rs199473145	0.0118/0.0/0.0078	14/120,204 (0.011)	25/245,708 (0.01)	VUS (BrS + stnQT)	VUS	VUS
*SLC4A3*	p.(Arg370His)	-	-	-	-	CM1717443	-	LP

Pathogenic and additional variants associated with Short QT Syndrome. DM: Disease Mutation, EVS: Exome Variant Server (EA: European-American, AA: African-American, All: All populations), ExAC: Exome Aggregation Consortium, gnomAD: Genome Aggregation Database, HGMD: Human Genome Mutation Database, MAF: Minor Allele Frequency, ACMG/AMP: American College of Medical Genetics and Genomics/Association for Molecular Pathology, ClinVar: Clinical Variation, P: Pathogenic, LP: Likely Pathogenic, VUS: Variant of Uncertain Significance, B: Benign, LQTS: Long QT Syndrome, SIDS: Sudden Infant Death Syndrome, BrS: Brugada Syndrome, AF: Atrial Fibrillation, stnQT: shorter than normal QT.

**Table 2:** Classification in silico of variants.

Gene	Protein	CADD	MetaSVM	GERP	M-CAP	MKL	MetaLR	MT	MA	PhyloP	PROVEAN	PPH2
*CACNA1C*	p.(Ala39Val)	N	N	NC	PD	D	N	DC	N	NC	N	PD
	p.(Gly490Arg)	N	N	NC	N	N	D	DC	N	NC	N	N
	p.(Asn547Ser)	N	D	NC	PD	N	D	DC	N	NC	N	B
	p.(Arg632Arg)	N	D	NC	D	D	D	DC	N	NC	N	N
	p.(Glu1115Lys)	N	D	NC	N	D	D	DC	N	NC	N	PD
	p.(Arg1780His)	N	D	NC	PD	N	D	DC	N	NC	N	N
	p.(Glu1829_Gln1833dup)	PD	D	C	D	D	D	DC	N	C	PD	PD
	p.(Arg1880Gln)	N	D	NC	N	D	D	DC	N	NC	N	N
	p.(Val2014Ile)	N	D	NC	N	D	D	DC	N	NC	N	B
	p.(Asp2130Asn)	N	N	NC	N	D	N	DC	N	NC	N	PD
*CACNA2D1*	p.(Ser755Thr)	N	N	NC	PD	D	N	DC	N	NC	PD	N
*CACNB2*	p.(Ser481Leu)	N	D	NC	N	D	D	DC	N	NC	N	N
*KCNH2*	p.(Glu50Asp)	PD	D	C	D	D	D	DC	N	C	PD	PD
	p.(Arg164Cys)	N	D	NC	D	D	D	DC	N	NC	N	N
	p.(Ile560Thr)	PD	D	NC	D	D	D	DC	PD	NC	PD	PD
	p.(Asn588Lys) c.(1764C>A)	PD	D	C	D	D	D	DC	N	C	N	PD
	p.(Asn588Lys)c.(1764C>G)	PD	D	C	D	D	D	DC	N	C	N	PD
	p.(Thr618Ile)	N	D	C	D	D	D	DC	PD	C	N	PD
	p.(Ser631Ala)	PD	D	C	D	D	D	DC	N	C	N	PD
	p.(Trp927Gly)	PD	D	NC	D	D	D	DC	N	NC	N	B
	p.(Arg1135His)	N	D	NC	D	D	D	DC	N	NC	N	B
*KCNJ2*	p.(Asp172Asn)	PD	D	NC	D	D	D	DC	N	NC	PD	PD
	p.(Glu299Val)	N	D	C	D	D	D	DC	N	C	PD	PD
	p.(Met301Lys)	PD	D	C	D	D	D	DC	N	C	PD	PD
	p.(Lys346Thr)	N	D	C	D	D	D	DC	N	C	PD	PD
*KCNQ1*	p.(Phe279Ile)	PD	D	C	D	PD	D	DC	N	C	D	PD
	p.(Val307Leu)	N	D	C	D	D	D	DC	PD	C	D	PD
	p.(Val141Met)	PD	D	C	D	D	D	DC	N	C	N	PD
	p.(Ile274Val)	N	N	NC	PD	D	N	DC	N	NC	PD	N
	p.(Arg259His)	N	D	NC	D	D	PD	DC	N	NC	N	B
*SCN5A*	p.(Arg689His)	N	D	NC	PD	D	PD	DC	N	NC	N	PD
*SLC4A3*	p.(Arg370His)	N	D	C	D	D	D	DC	N	C	D	PD

Classification in silico of variants. Combined Annotation Dependent Depletion -CADD-, Genomic Evolutionary Rate Profiling -GERP-, MetaSVM, Mendelian Clinically Applicable Pathogenicity Score -M-CAP-, Fathmm-MKL, MetaLR, Mutation Taster -MT-, Mutation Assessor -MA-, Phylogenetic Analysis -PhyloP-, Protein Variation Effect Analyzer -PROVEAN-, Polymorphism Phenotyping v2 -PolyPhen2, PPH2-, DC: Disease Causing, B: Benign, D: Deleterious/Damaging, PD: Probably Damaging/Deleterious, N: Neutral, C: Conserved aminoacid, NC: No conserved aminoacid.

**Table 3:** Classification of variants following American College of Medical Genetics and Genomics/Association for Molecular Pathology (ACMG/AMP) criteria.

Gene	Protein	Population Data	Computational and Predictive Data	Functional Data	Segregation Data	De novo Data	Allelic Data	Other Database	Other Data	ACMG/AMP Classification
*CACNA1C*	p.(Ala39Val)	PM2	C	PP2	NA	PM6	NA	C	NA	VUS
	p.(Gly490Arg)	BS1	C	PP2	NA	NA	NA	BP6	NA	VUS
	p.(Asn547Ser)	PM2	C	PP2	NA	PM6	NA	C	NA	VUS
	p.(Arg632Arg)	PM2	C	PP2	NA	PM6	NA	C	NA	VUS
	p.(Glu1115Lys)	PM2	C	PP2	NA	NA	NA	C	NA	VUS
	p.(Arg1780His)	PM2	C	PP2	NA	PM6	NA	C	NA	VUS
	p.(Glu1829_Gln1833dup)	PM2	PP3	PP2	NA	NA	NA	C	NA	VUS
	p.(Arg1880Gln)	PM2	C	PP2	NA	PM6	NA	C	NA	VUS
	p.(Val2014Ile)	PM2	C	PP2	NA	PM6	NA	C	NA	VUS
	p.(Asp2130Asn	PM2	C	PP2	NA	NA	NA	C	NA	VUS
*CACNA2D1*	p.(Ser755Thr)	BS1	C	BS3	NA	NA	NA	C	NA	B
*CACNB2*	p.(Ser481Leu)	PM2	C	PS3	NA	NA	NA	C	NA	VUS
*KCNH2*	p.(Glu50Asp)	PM2	PP3	PP2	NA	PM6	NA	C	NA	LP
	p.(Arg164Cys)	PM2	C	PP2	NA	NA	NA	C	NA	VUS
	p.(Ile560Thr)	PM2	PP3	PS3	NA	PM6	NA	C	PP4	P
	p.(Asn588Lys) c.(1764C>A)	PM2	PP3	PS3	NA	PM6	NA	PP5	PP4	P
	p.Asn588Lys c.(1764C>G)	PM2	PP3	PS3	NA	PM6	NA	PP5	PP4	P
	p.(Thr618Ile)	PM2	PP3	PS3	NA	PM6	NA	C	PP4	P
	p.(Ser631Ala)	PM2	PP3	PS3	NA	PM6	NA	C	NA	LP
	p.(Trp927Gly)	PM2	PP3	PP2	NA	PM6	NA	C	NA	LP
	p.(Arg1135His)	PM2	C	PS3	PP1	NA	NA	C	NA	VUS
*KCNJ2*	p.(Asp172Asn)	PM2	PP3	PS3	NA	PM6	NA	PP5	PP4	P
	p.(Glu299Val)	PM2	C	PS3	NA	PM6	NA	C	PP4	P
	p.(Met301Lys)	PM2	PP3	PS3	NA	PM6	NA	C	C	P
	p.(Lys346Thr)	PM2	C	PP2	NA	PM6	NA	C	C	VUS
*KCNQ1*	p.(Phe279Ile)	PM2	PP3	PP2	NA	PM6	NA	C	NA	LP
	p.(Val307Leu)	PM2	PP3	PS3	NA	PM6	NA	PP5	NA	P
	p.(Val141Met)	PM2	PP3	PS3	PP1	PS2	NA	PP5	PP4	P
	p.(Ile274Val)	BS1	C	PP2	NA	NA	NA	C	NA	VUS
	p.(Arg259His)	PM2	C	PS3	NA	NA	NA	C	NA	VUS
*SCN5A*	p.(Arg689His)	PM2	C	PS3	NA	PM6	NA	C	C	VUS
*SLC4A3*	p.(Arg370His)	PM2	PP3	PS3	NA	PM6	NA	C	PP4	LP

Classification of variants following American College of Medical Genetics and Genomics/Association for Molecular Pathology (ACMG/AMP) criteria. B: Benign, BA: Evidence of benign impact Stand-alone, BP: Evidence of benign impact Supporting, BS: Evidence of benign impact Strong, C: Contradictory data, LP: Likely Pathogenic, NA: Not available data, P: Pathogenic, PM: Evidence of Pathogenicity Moderate, PP: Evidence of Pathogenicity Supporting, PS: Evidence of Pathogenicity Strong, PVS: Evidence of Pathogenicity Very Strong, VUS: Variant of Uncertain Significance.
